# The Diverticular Disease Registry (DDR Trial) by the Advanced International Mini-Invasive Surgery Academy Clinical Research Network: Protocol for a Multicenter, Prospective Observational Study

**DOI:** 10.29337/ijsp.157

**Published:** 2021-08-30

**Authors:** Matteo Origi, Pietro Achilli, Giacomo Calini, Andrea Costanzi, Michela Monteleone, Isacco Montroni, Dario Maggioni, Eugenio Cocozza, Stefano Megna, Mauro Totis, Nicolo’ Tamini, Antonio Ziccarelli, Gaetano Filippone, Giovanni Ferrari, Jacopo Crippa, Antonino Spinelli, Giulio M. Mari, on behalf of the AIMS Academy Clinical Research Network

**Affiliations:** 1 General Surgery Department, Niguarda Hospital, Milano, Italy; 2 Department of Medical Area (DAME), University of Udine, Udine, Italy; 3 General Surgery Department, Merate Hospital, ASST Lecco, Italy; 4 General Surgery Department, Ospedale degli Infermi, Faenza, Italy; 5 General Surgery Department, Desio Hospital, ASST Brianza, Italy; 6 General Surgery Department, ASST Settelaghi, Italy; 7 General Surgery Department, ASST Monza, Italy; 8 General Surgery Department, ASUFC, Udine, Italy; 9 Humanitas Research Hospital, IRCCS, Rozzano – Milano, Italy; 10 Department of Biomedical Sciences, Humanitas University, Pieve Emanuele – Milano, Italy

**Keywords:** diverticular disease, diverticulitis, diverticulosis, clinical protocols, multicenter study, prospective study

## Abstract

Diverticular disease is an increasingly common issue, with a variety of clinical presentations and treatment options. However, very few prospective cohort studies explore outcomes between the different presentations and treatments. The Diverticular Disease Registry (DDR Trial) is a multicenter, prospective, observational cohort study on behalf of the Advanced International Mini-Invasive Surgery (AIMS) academy clinical research network. The DDR Trial aims to investigate the short-term postoperative and long-term quality of life outcomes in patients undergoing surgery or medical treatments for diverticular disease. DDR Trial is open to participation by all tertiary-care hospitals. DDR Trial has been registered at ClinicalTrials.gov (NCT 04907383). Data collection will be recorded on Research Electronic Data Capture (REDCap) starting on June 1^st^, 2021 and will end after 5 years of recruitment. All adult patients with imaging-proven colonic diverticular disease (i.e., symptomatic colonic diverticulosis including diverticular bleeding, diverticulitis, and Symptomatic Uncomplicated Diverticular Disease) will be included. The primary outcome of DDR Trial is quality of life assessment at 12-month according to the Gastrointestinal Quality of Life Index (GIQLI). The secondary outcome is 30-day postoperative outcomes according to the Clavien-Dindo classification. DDR Trial will significantly advance in identifying the optimal care for patients with diverticular disease by exploring outcomes of different presentations and treatments.

**Highlights:**

- Diverticular disease (i.e., diverticulitis, bleeding) has different treatments.
- This is a clinical protocol for the Diverticular Disease Registry (DDR Trial).
- DDR Trial is a multicenter, prospective, observational cohort study open to participation.
- DDR Trial will study short-term postoperative and long-term quality of life outcomes.
- Medical treatments, interventional radiology and surgery will be explored.

## Introduction

Diverticular Disease (DD) is defined as clinically significant and symptomatic colonic diverticulosis and includes diverticular bleeding, diverticulitis, and Symptomatic Uncomplicated Diverticular Disease (SUDD) [1]. DD prevalence increases significantly with age, ranging from 10–20% in those younger than 40 years up to 50 to 70% in those older than 70–80 years [2,3]. While diverticulitis occurs in around 5% (1.7% complicated) of patients with diverticulosis [4], admission rate is steadily increasing overall and at a higher rate in younger patients [5]. Indeed, risk factors for DD such as obesity, diet low in fiber, lack of exercise, smoking, and aging of population are increasing. Along with the different clinical presentations and populations affected by DD, diagnosis and treatments have consistently changed. To standardize the treatments, several classifications have been proposed through the years, and several algorithms developed focusing on clinical features and patient’s quality of life more than anatomical and pathological landmarks [1,2,6,7,8]. Moreover, different surgical treatments have been proposed [8,9,10,11,12], while the surgical approach has been shifting to a minimally invasive approach even in the emergency setting [13,14]. Indeed, population, natural history, presentation, severity, and treatment for DD are heterogeneous, and optimal care for patients with DD needs high-quality evidence from large cohort studies [15]. Initial experiences of registry on DD recently appeared in the literature [16,17,18], suggesting the genesis of networks dedicated to DD as the best feasible way to strengthen clinical evidence. However, very few prospective cohort studies explore the outcomes between the different presentations and treatments for DD.

The Diverticular Disease Registry (DDR Trial), on behalf of the Advanced International Mini-Invasive Surgery (AIMS) academy clinical research network, will investigate both short-term 30-day postoperative outcomes and 12-months quality of life assessment in patients with DD. All different surgical and medical treatments will be explored to define the best management for each different presentation of DD. In addition, clinical parameters correlating with favorable outcomes in patients undergoing surgery or medical treatment for DD will be considered according to recurrence, health utilization, and mortality rates.

## Methods

### Study design

This protocol describes a multicenter, prospective, observational cohort study on diverticular disease. The DDR Trial is open to participation by all tertiary-care hospitals. The DDR Trial aims to investigate short-term postoperative and long-term quality of life outcomes in patients undergoing surgery or medical treatments for diverticular disease. The primary outcome is the quality of life assessment at 12-month in patients with diverticular disease according to the Gastrointestinal Quality of Life index (GIQLI) [19]. The secondary outcome is 30-day postoperative outcomes according to the Clavien-Dindo classification [20].

### Study population

After written informed consent, all consecutive patients encountered or admitted to a General Surgery or Gastroenterology Unit of a tertiary-care hospital with a diagnosis of DD will be enrolled in the DDR Trial. One investigator in each center, is responsible to preserve the informed consents, to update data collection, and to follow-up the patients through their medical record numbers. Patients will be enrolled in the DDR Trial starting from June 2021 and ending after 5 years of recruitment, according to the inclusion criteria.

#### Inclusion criteria

All adult patients (≥18 years old) with colonic DD will be included. DD is defined as clinically significant and symptomatic colonic diverticulosis and includes diverticular bleeding, diverticulitis, and Symptomatic Uncomplicated Diverticular Disease (SUDD). Symptoms include but are not limited to fever, alternating bowel habits, pain, diverticular bleeding, and dysuria. The presence of colonic diverticulosis needs to be confirmed by imaging or during emergency surgery and/or by pathological diagnosis. After obtaining written informed consent, either patient presenting in an outpatient or emergency department will be enrolled. According to the European Society of Coloproctology guidelines [1], a colonoscopy confirming DD will be required during the follow-up or before surgical treatment, if feasible.

#### Exclusion criteria

Patients with asymptomatic diverticulosis only (imaging or colonoscopy incidental finding of colonic diverticula without symptoms, evidence of acute inflammation or complication), with other concomitant bowel diseases (inflammatory bowel disease, colorectal cancer, and ischemic bowel), or without informed consent will be excluded.

#### Patients enrollment

Following patient enrollment, study variables will be collected as patients progress through routine care pathways. Patients’ presentation and treatment may range from mild DD to peritonitis and from non-antibiotic treatment to surgery, respectively. Every patient’s presentations and treatment regimens will be recorded with the appropriate case report forms (CRF).

### Study variables

The DDR Trial’s variables will include patient baseline, presentation, diagnosis, treatment, and follow-up of patients enrolled, as reported in the CRF. Study variables will be documented for every patient’s presentations and treatments.

Patients enrollment and baseline will be recorded during the first surgical visit, together with a complete clinical examination. In particular, anthropometrics, habits, comorbidity, relevant medications, and baseline quality of life will be collected and categorized (CRF1- CRF2). Charlson comorbidity score adjusted for age will be calculated for every patient [21]. Patients older than 70 years old will be assessed for frailty risk using the modified Frailty Index described by Robinson et al. [22].

Patients and disease presentations are collected according to two different settings (from the emergency room or ambulatory/outpatient clinic). Symptoms, previous presentation, diagnosis, and treatments, need for admission, and treatment will be recorded (CRF3).

Instrumental diagnostic data from colonoscopy, abdominal computerized tomography (CT) and virtual CT colonoscopy be collected (CRF4).

Surgical pre- and intraoperative details will be recorded in the DDR Trial both for emergency procedures and for elective procedures, such as surgical setting, surgical procedure, resection, peritoneal lavage, laparoscopy, operative time, estimated blood loss, conversion, type of mesenteric artery ligation, stapler used and other technical aspects (CRF5). 30-day postoperative complications will be reported and graded according to the Clavien-Dindo classification [20]. Length of stay and post-discharge complications will be evaluated and recorded. Application of Enhanced Recovery After Surgery (ERAS) protocols will be considered valid if only at least 80% of the designed colorectal items will be satisfied [23].

Specimen length, the presence of microscopic and macroscopic abscesses, Crohn’s-like reaction, lymphocyte infiltration, and unknown cancer will be obtained by the pathology report for patients who underwent surgical resection (CRF6).

Follow-up will be done yearly assessing patient status, time to recurrence, symptoms, medication, consideration of surgical intervention, and quality of life (CRF7). Quality of life will be evaluated according to the Gastrointestinal Quality of Life index (GIQLI) [19]. The GIQLI is a 36-item patient reported outcomes instrument designed to assess GI-specific health-related quality of life in clinical practice and clinical trials of patients with GI disorders. It has five domains (GI symptoms, emotion, physical function, social function and medical treatment) and subscores range from 0–4 while the total score range from 0–144. Higher scores mean better GI health-related quality of life [19]. In addition, Female Sexual Function Index (FSFI) [24], International Consultation on Incontinence Questionnaire (ICIQ) [25], International Index of Erectile Function (IIEF) [26,27], and International Prostate Symptom Score (IPSS) [28], will be used, as appropriate.

### Data management

The Diverticular Disease Registry is managed using Research Electronic Data Capture (REDCap), a secure web-based platform designed to support databases for research studies [29,30]. Data will be prospectively uploaded in the Diverticula Disease Registry by the local principal investigator for each hospital via personal REDCap accounts. Quarterly meetings will be set between the study coordinators and the participating centers. The local principal investigator might be assisted by one or more collaborators according to the volume of the uploaded data. One investigator in each center will be responsible for the follow-up data. A data manager (AP) will regularly control the quality of the data provided, missing or implausible data will be identified and addressed.

### Data analysis plan

Data analysis will primarily explore the quality of life in patients with DD after different treatments. Secondly, we aim to evaluate surgical management and short-term 30-day postoperative outcomes. We will explore baseline covariants that may identify clinical parameters correlating with favorable outcomes or be predictive of failure in patients undergoing different treatments for DD. Furthermore, we will evaluate recurrence, successive presentations (health utilization), and mortality rates. No personal or hospital-specific comparisons will be performed. The results of the DDR Trial will be reported according to the STROBE statement [31].

#### Statistical analysis

Categorical data will be summarized by frequencies and percentages, and compared using the chi-squared test. Continuous data will be summarized by mean and standard deviation (normally distributed data) or median and interquartile range (non-normally distributed data), and will be compared using the t-test or ANOVA (normally distributed data) and Mann-Whitney U test or Kruskal-Wallis test (non-normally distributed data), as appropriate. Logistic regression models will be developed to examine the relationship between outcomes and covariates. Survival analysis (e.g., Kaplan-Meier) will be used to study the time-to-event. A two-sided p-value <0.05 will be considered statistically significant.

### Ethics

Institutional Review Board approval was obtained by the Comitato Etico Milano Area 3 (approval number 233-22042021). The study protocol has been registered as an observational study at ClinicalTrials.gov (NCT04907383). Every local principal investigator should request approval from the local ethics committee. No sensitive or identifiable data will be collected in the DDR Trial on the REDCap database. However, every local principal investigator should preserve a connection between the patient ID and the unidentified code for up to 5 years from the enrollment date for future studies. Patients enrollment will be subordinate to formal informed consent. The present study does not require a change in clinical practice, additional procedures, imaging, or lab test. Follow-up includes an oral assessment with standardized questionnaires, and it will be carried out by the local principal investigator and collaborators. The study will be carried out in accordance with the Declaration of Helsinki. Non-respondent and loss of follow-up will be reported.

### Funding

This research did not receive any specific grant from funding agencies in the public, commercial, or not-for-profit sectors. No financial reimbursement will be provided to the participating centers.

### Study dissemination

The DDR Trial is on behalf of the Advanced International Mini-Invasive Surgery (AIMS) academy clinical research network. The results of the DDR Trial will be published in a peer-reviewed biomedical journal. Because of the multicentric nature of the study, it might be presented at national and international meetings. The DDR Trial is open to participation by all tertiary-care hospitals. Chief investigators and collaborators are responsible for calling for participation. All participants will have access to the data collected and use them to design further studies and write scientific articles. The AIMS Academy will host data, and all publications using data from the DDR Trial will be coordinated and reviewed by the chief investigators.

### Authorship policy

Since the success of the study depends on a larger number of healthcare providers, credit for the results will be given to all the healthcare providers who have collaborated and participated in the study. For every tertiary-care hospital participating in the study, the local principal investigator might include one or more collaborators according to the volume of the data uploaded. The head authorship in the publications of the primary results will include the chief investigators on behalf of the Advanced International Mini-Invasive Surgery (AIMS) academy clinical research network and all the collaborative groups. Authorship of additional papers will be accorded with the chief investigators.

## Discussion

Although DD is a common condition, we continue to struggle in defining optimal care as DD is still poorly understood. The primary aim of the DDR Trial is to prospectively collect data on patients with DD, focusing on the clinical presentation, diagnosis, treatments, and clinical follow-up.

Standardizing the data collection of patients with diverticulitis is often tricky for clinical and logistic reasons. Indeed, patients with DD have different healthcare accesses: emergency department, endoscopic unit, surgical, and gastroenterological outpatient departments. Moreover, these patients might be admitted to a surgical or gastroenterological ward. As a consequence, the multiplicity of clinical scenarios results in a lack of accuracy in generating a register that collects clinical data. Herein, we want to pave the way to a collaborative, comprehensive, and user-friendly tool that aims to provide prospective clinical data.

Reaching scientific evidence about medical and surgical treatment of DD is extremely demanding, and it requires considerable resources. Therefore, high-quality evidence is limited, and frequently recommendations have low levels of evidence. Moreover, the decision-making process for DD treatment is determined on a case-by-case basis according to the different clinical presentations, settings, surgeon expertise, and patient preferences [15]. In addition, the decision for elective surgery for SUDD or smouldering diverticular disease (SDD) truly belongs to a clinical grey zone that needs to be clarified. Overall, because these clinical nuances are frequently present in daily practice and so hardly intelligible, it becomes clear the need for a structured outcome-related prospective registry. Indeed, follow-up data for patients with SUDD o SDD, either after surgical or conservative treatments, will provide high-quality evidence for management recommendations and practical decision making.

As previously mentioned, other similar experiences are now ongoing in the medical panorama. Although conceived as snapshot studies, the REMAD registry has produced consistent and valuable data for clinical practice from patient follow-up [16,17], and the DAMASCUS study will investigate current practice and outcomes worldwide [18]. Differently, after the primary study at 12-month, the DDR Trial will have a recruitment period of 5 years with the aim of collecting long-term prospective outcomes for both surgical and medical treatment from a large cohort of patients.

## Conclusions

As stated by the present protocol, the DDR Trial will establish a multicenter, prospective, observational cohort study for patients with diverticular disease based on systematic data collection in order to increase knowledge on diverticular disease. By exploring outcomes of different presentations and treatments in the daily surgical and clinical practice, DDR Trial will significantly advance in identifying the optimal care for patients with diverticular disease.

## Additional file

The additional file for this article can be found as follows:

10.29337/ijsp.157.s1 Case Report Forms. Study variables are reported in detail.
