# Supplementary material for: The Diverticular Disease Registry (DDR Trial) by the Advanced International Mini-Invasive Surgery Academy Clinical Research Network: Protocol for a Multicenter, Prospective Observational Study
Source: Int J Surg Protoc. 2021 Aug 30;25(1):194–200. doi: 10.29337/ijsp.157 (PMC8415185; doi:10.29337/ijsp.157)
Supplement: Case Report Forms. — Study variables are reported in detail. [file ijsp-25-1-157-s1.pdf]

**Case Report Forms**

| |
|---|
| **CRF1: ENROLLMENT** |
| Patient ID, informed consent Y/N, unidentified patient code, date of enrollment, center of enrollment. |
| **CRF2: PATIENTS BASELINE** |
| Patients sex, age, weight, height, BMI, Charlson Comorbidity score [21], Robinson score [22], Details of previous abdominal surgery, smoke, alcohol, fiber intake, constipation, physical activity, dislipidemia, diabetes, NSAIDs, steroids, immunosoppressant, ASA score, baseline QoL scores. |
| **CRF3: PRESENTATION** |
| *from Emergency Room* |
| Symptoms: fever, alternating bowel habits, pain, diverticular bleeding, dysuria<br>Previous emergency room presentation, hospital admission, length of stay, previous diagnosis.<br>Laboratory test: ESR, WBC, CRP, fecal calprotectine. Previous treatment with Mesalazine, duration.<br>Diagnosis: un/complicated diverticulitis, SUDD, recurrence, smouldering diverticulitis.<br>No hospital admission: follow-up, delayed elective surgery.<br>Hospital admission: emergency surgery, conservative treatment, percutaneous drainage. |
| *from Ambulatory* |
| Symptoms: fever, alternating bowel habits, pain, diverticular bleeding, dysuria<br>Previous presentations and hospital admission, length of stay. Previous diagnosis: SUDD (GIQLI), recurrence (n.), smouldering diverticulitis (GIQLI), complicated diverticulitis (drainage), years of follow-up.<br>Previous treatment with Mesalazine, duration. Indication: follow-up, antibiotic, surgery, delayed surgery. |
| **CFR4: INSTRUMENTAL DIAGNOSTIC** |
| *Colonoscopy* |
| Completed, location of DD, inflammation, bleeding, mucosal changes, IBD, secondary location of DD |
| *CT-scan* |
| Timing, use of IV or rectal contrast, colonic wall thickening, pericolic fat stranding, location of multiple diverticula and of inflamed diverticula, abscess's location and dimension, free extraluminal gas, fluid or contrast extravasation, colovescical, colovaginal or coloenteric fistula, obstruction, suspected cancer. |
| *Virtual CT-colonoscopy* |
| location of DD, secondary colonic location, extenction, wall rigidity, stenosis, dolichocolon |
| *Classification* |
| Hinchey modified (I - IIa - IIb - III - IV) [32] , AAST grading system (I - II - III - IV - V) [33] |
| **CFR5: SURGERY** |
| *Pre- and intraoperative details* |
| Emergent or elective setting, previous treatments (percutaneous drainage, laparoscopic lavage), surgical resection, operative time, laparoscopic, cause of conversion, vessel ligation, colon mobilization, energy device, cartridge, anastomosis details, estimated blood loss, surgical second look, stoma type and closure. |
| *Postoperative outcomes* |
| 30-day complications, Clavien-Dindo classification, anastomotic leak, bleeding, obstruction, wound infection, medical complication, ERAS compliance, length of stay. |
| **CFR6: PATHOLOGY** |
| Length of specimen, presence of abscess (microscopic, macroscopic), Crohn's-like reaction, lymphocyte infiltration, presence of unknown cancer |
| **CFR7: FOLLOW-UP** |
| Patient status, recurrence, time to recurrence, symptoms: fever, alternating bowel habits, pain, diverticular bleeding, dysuria; QoL scores (GIQLI, IPSS, ICIQ, FSFI, IIEF) at 12 months, need of medication, consideration of redo/surgery. |
| **CFR8: END** |
| Complete follow-up, last date of follow-up, reason for withdrawal, patient status. |

Abbreviation: AAST: American Association for the Surgery of Trauma; ASA: American Society of Anesthesiologists; BMI: body mass index; CRP: C-reactive protein; DD: diverticular disease; ER: emergency room; ERAS: Enhanced Recovery After Surgery; ESR: erythrocyte sedimentation rate; FSFI: Female Sexual Function Index [24]; GIQLI: Gastrointestinal Quality of Life index [19]; IBD: inflammatory bowel disease; ICIQ International Consultation on Incontinence Questionnaire (ICIQ) [25]; IIEF: International Index of Erectile Function [26, 27]; IPSS: International Prostate Symptom Score[28]; NSAIDs: non-steroidal anti-inflammatory drugs; QoL: quality of life; SUDD: symptomatic uncomplicated diverticular disease; WBC: white blood cell.

### References

32. **Sher ME, Agachan F, Bortul M, Nogueras JJ, Weiss EG, Wexner SD.** Laparoscopic surgery for diverticulitis. *Surg Endosc*. 1997; 11(3): 264–7. DOI: *https://doi.org/10.1007/s004649900340*

33. **Shafi S, Priest EL, Crandall ML,** et al. Multicenter validation of American Association for the Surgery of Trauma grading system for acute colonic diverticulitis and its use for emergency general surgery quality improvement program. *J Trauma Acute Care Surg*. 2016; 80(3): 405–10; discussion 10–1. DOI: *https://doi.org/10.1097/TA.0000000000000943*
